# A Tubular Biomaterial Construct Exhibiting a Negative Poisson’s Ratio

**DOI:** 10.1371/journal.pone.0155681

**Published:** 2016-05-27

**Authors:** Jin Woo Lee, Pranav Soman, Jeong Hun Park, Shaochen Chen, Dong-Woo Cho

**Affiliations:** 1 Department of Molecular Medicine, School of Medicine, Gachon University, 7-45, Songdo-dong, Yeonsu-ku, Incheon, 406-840, Republic of Korea; 2 Department of Biomedical and Chemical Engineering, Syracuse University, 318 Browne Hall, Syracuse, NY, 13244, United States of America; 3 Department of Mechanical Engineering, Pohang University of Science and Technology (POSTECH), San 31, Hyoja dong, Nam-gu, Pohang, Gyeongbuk, 790-781, Republic of Korea; 4 Department of NanoEngineering, University of California San Diego, 9500 Gilman Drive, Atkinson Hall, MC-0448, La Jolla, CA, 92093, United States of America; University of Manchester, UNITED KINGDOM

## Abstract

Developing functional small-diameter vascular grafts is an important objective in tissue engineering research. In this study, we address the problem of compliance mismatch by designing and developing a 3D tubular construct that has a negative Poisson’s ratio ν_xy_ (NPR). NPR constructs have the unique ability to expand transversely when pulled axially, thereby resulting in a highly-compliant tubular construct. In this work, we used projection stereolithography to 3D-print a planar NPR sheet composed of photosensitive poly(ethylene) glycol diacrylate biomaterial. We used a step-lithography exposure and a stitch process to scale up the projection printing process, and used the cut-missing rib unit design to develop a centimeter-scale NPR sheet, which was rolled up to form a tubular construct. The constructs had Poisson’s ratios of -0.6 ≤ ν_xy_ ≤ -0.1. The NPR construct also supports higher cellular adhesion than does the construct that has positive ν_xy_. Our NPR design offers a significant advance in the development of highly-compliant vascular grafts.

## Introduction

In most developed countries, the number of patients with cardiovascular diseases is growing, and the associated health care cost has become a serious socio-economic burden [1, 2]. In the United States, more than 500,000 surgical operations for cardiovascular diseases have been performed annually [3]. Although auto-transplantation is an ideal solution to replace damaged blood vessels, not all patients can offer suitable blood vessels for surgery. Furthermore, this method needs an additional surgery to obtain suitable vessels. Due to the shortage of autologous vessels, demands for non-autologous vessels have increased. As a result, synthetic replacements including expanded polytetrafluorethylene (PTFE) grafts and Dacron have been invented and used as large-diameter blood vessel substitutes. However, artificial grafts for small-diameter (< 6 mm) blood vessel (SDBV) substitutes have not yielded successful clinical outcomes. Many challenges remain, including infection, acute thrombogenicity of the graft, early occlusion, anastomotic intimal thickening, formation of aneurysms, and atherosclerotic disease [4–12].

Tissue engineering could provide a promising method to develop successful SDBVs by using a synthetic substitute matrix in combination with living cells and biomolecules. Some previous research to develop functional SDBV replacement grafts has obtained decellularized material from vascular or non-vascular tissues, then used it as a scaffold to regenerate SDBVs, but decellularization damages the tissue and reduces its tensile strength [13–20]. Another approach is to develop a tissue-engineered graft that consists of cell sheets wrapped on a porous tubular construct [21–25]. However, their works did not match a compliance between the native vessels and SDBVs graft. A compliance, which is called an elasticity, is the property of a body that enables it to resume its original size or shape when a distorting force is removed. The compliance is assessed by the amount of force per unit area to achieve a given amount of deformation. Because a compliance mismatch between implanted graft and original vessel generates problems of blood stream including a turbulence, artificial grafts cannot easily be applied successfully to small-diameter vessels [26].

Several biodegradable polymer constructs, including polyhydroxyalkanoate, Poly(glycolic acid) (PGA), poly(lactic acid) (PLA), poly-4-hydroxybutyrate, poly(caprolactone)–co-poly(lactic acid) (PCL-co-PLA) and poly(ethylene glycol) (PEG) have been investigated for application for vascular tissue regeneration [27–34], but most constructs were implanted to the low-pressure pulmonary circulation of 20 to 30 mm Hg due to their compliance mismatch, which often causes anastomotic intimal hyperplasia.

Poisson’s ratio is [35]

$$\nu_{\text{xy}}=\;-\;\varepsilon_{\text{y}}/\varepsilon_{\text{x}}$$ (1)

Where ε_y_ transverse strain caused by an axial strain ε_x_. That is to say, Poisson's ratio is the ratio of transverse contraction (expansion) strain to longitudinal expansion (contraction) strain in the direction of tensional (compressional) force [36]. A lower case Greek nu(ν), Poisson's ratio coefficient, contains a minus sign so that normal materials have a positive ratio. Generally, when a material is compressed in one direction it expands in the two directions perpendicular to that direction, and when stretched it contracts in the other directions; this characteristic is a positive Poisson’s ratio ν_xy_ (PPR). However, some materials that have unusual molecular structures display a negative Poisson’s ratio (NPR). At the NPR structure, by the tension, both longitudinal expansion and transverse expansion are observed. And both longitudinal contraction and transverse contraction are observed by a compression. Examples include some crystalline materials, carbon allotropes, foams and polymers and laminates [37]. This unusual characteristic may present the possibility of connecting vascular graft harmoniously to existing tissue by using a material with NPR in the implanted graft. Because a biomaterial graft should be tested in a high-pressure environment such as the coronary artery, compliance matching is a significant factor in the design of an artificial graft. For this reason, modulation of NPR is a possible solution to match the product compliance. In this work, we develop a highly-compliant small-diameter blood vessel substitute by imparting an NPR to a biocompatible material.

Arterial endothelium has NPR [38–41]. Especially, the arterial endothelium is exposed to a wall shear stresses as well as to a cyclic circumferential strain because of the pulsatile flow of blood. The thickness of the axially aligned sub-endothelial fiber layers in bovine carotid arteries increased during circumferential strain; this result is consistent with this layer having NPR [42]. A vascular graft with NPR would be compressed and stretched by the pressure fluctuation caused by the pulsatile flow. Such a NPR would enable simultaneous stretching in the axial and transverse directions, and would integrate with native tissues and promote regeneration of new tissue better than do existing constructs.

Therefore, we designed a tubular construct with NPR by assembling a cut-missing rib pattern. We used a 3D printing system based on a digital micro-mirror device (DMD) to fabricate a construct from a hydrogel based on poly(ethylene glycol) diacrylate (PEGDA). To fabricate a large sample we used a step-and-print approach to scale up the 3D printing process. By stitching adjacent patterns, we built a large patch with the NPR trait, then rolled up the patch to form a tubular NRP graft.

## Materials and Methods

### Preparation of Photo-crosslinkable Monomer

To fabricate the hydrogel based construct, we mixed PEGDA (Mn700, Sigma-Aldrich, St. Louis, MO, USA) and acrylic acid (AA, Sigma-Aldrich, St. Louis, MO, USA), then added 1.25% (w/v) of a photoinitiator (Irgacure 2959, CIBA Chemicals, Basel, Switzerland) to the mixture. We also added 0.25% (w/v) of a UV-absorbing agent TINUVIN 234 (CIBA Chemicals, Basel, Switzerland) to control the thickness of the microstructures by reducing the curing depth. Finally, we added 0.02% (w/v) of a free-radical quencher, (4-hydroxy-2, 2, 6, 6-tetramethylpiperidine 1-oxyl, TEMPO, Sigma-Aldrich, St. Louis, MO, USA) to improve the contrast of UV-curing process and to optimize the resolution of the feature.

### Projection based 3D Printing

**Fig 1:**
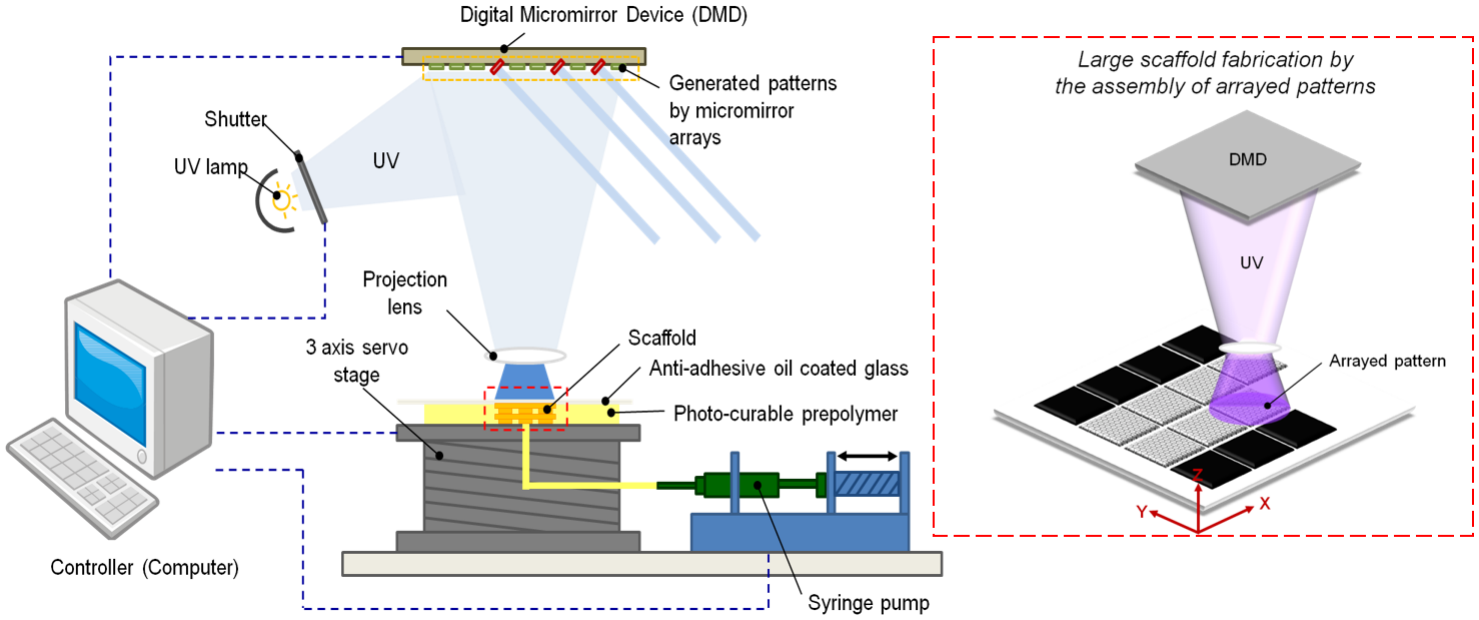
(Left) Schematic diagram of a projection based 3D printing system with a digital micro-mirror device (Right) Schematic showing the step-and-print process for scale-up printing.

To fabricate the NPR and PPR patterns in the hydrogel, we integrated a DMD-based 3D printing system with x-y-z axes (Fig 1). First, we used computer-aided design (CAD) software (AutoCAD, Autodesk Inc., San Raphael, CA, USA) to design the 2D model of an unrolled whole pattern, and the transformed the model to a bitmap image. We sized each image to 1920 x 1080 pixels to match the number of mirrors of the DMD system. Then, we exported the separated images to LabVIEW software (National Instruments (NI), Austin, TX, USA). We used LabVIEW to control each mirror in the DMD. We used the bitmap graphics files as virtual photo-masks for a photo-crosslinking process. A closed-loop stage was placed 100 μm under a transparent quartz plate to leave a gap of 100 μm between the stage base and the plate. Then a micro-pump was used to inject 150 μl of photo-crosslinkable pre-polymer into the gap. UV light for polymerization was spatially modulated by the DMD, which was operated using virtual masks controlled by the software. The UV light passes through a projection lens, then strikes a projection plane which is the lower surface of the quartz substrate. The pre-polymer solution was exposed to a UV dosage of 50 mW/cm^2^ for 3.5 s to solidify selected locations of the pre-polymer. After polymerization, the X and Y axis stages moved the projection plane to a new position to build a new part. The new part was stitched to the previous fabricated part. The process was repeated to achieve a desired large sample. A bottom side of the quartz plate which was coated with a silane (tridecafluoro-1, 1, 2, 2-tetrahydrooctyl-1 tri-chlorosilane) (United Chemical Technologies, Bristol, PA, USA) that reduces the surface energy helped the releasing process [43, 44].

### Deformation Simulation using a Finite Element Analysis

To design the 2D construct with unit-cell models, AutoCAD was utilized. Unit-cell structures were designed based on the literature, then imported into Solidworks 2009 (SolidWorks Corp., Concord, MA, USA). To make a unit-cell-patterned tubular construct, we engraved the 2D pattern on the thin tube [38]. The 3D models were used in finite element analysis (FEA) to simulate the deformation of PEGDA-based 3D constructs.

To execute the FEA analysis, we used the elastic modulus of 7.06 MPa and a density of 1.12 g/ml acquired in previous work for the PEGDA-based biomaterial [45]. The simulations result instructed us whether unit-cell constructs could yield NPR theoretically as we desired. The simulations were performed before the experiments. In brief, one side of the tube was fixed, then a tensile strain in the axial direction was applied to its other side. These simulations were conducted for unit cells of both cut-missing-rib (NPR) and intact-rib (PPR).

### Mechanical testing

To acquire the stress-strain plot of the construct, a tensile test was performed using universal testing machine (UTM, Instron, Norwood, MA, USA). Samples were linked with two tensile holders and were loaded at a strain rate of 3mm/min. Stress-strain curves were plotted and analyzed using a Microsoft Excel to facilitate the calculation of the testing parameters.

### Strain Test to Determine the Poisson’s Ratios

To measure ν_xy_ of the two types of tubular constructs, strain tests were conducted. The PEGDA construct was loaded into an in-house strain measurement system. One end of this system was fixed to an immovable stage with a polyethylene pipe and the other end was fixed to a nano-positioning stage of a single-axis. The stage is linked to a motorized servo actuator (CMA-25CCCL, Newport Corp., Irvine, CA, USA) that can provide 200-nm incremental motion. The actuator is controlled by a motion controller (ESP300 Axis Motion Controller, Newport Corp., Irvine, CA, USA). A tensile stress in the axial direction by the pulling motion was transferred to the end of a PEGDA tubular construct that is attached to the movable stage, thereby imposing axial strain. The transverse and axial movements of the construct were recorded using a color CCD camera system (CV-S3200P, JAI Inc., San Jose, CA, USA) and a magnification lens (Edmund Industrial Optics, Barrington, NJ, USA). Strains in the transverse and axial directions were estimated by measuring the results of the displacement. Image digitalizing software (GetData Graph Digitizer 2.24) was used to digitize the optical images to enable calculation of an accurate deformation from the optical image set.

### Calculation of Poisson’s Ratios

The overall transverse deformation of the constructs caused by axial strains was used to calculate ν_xy_ (Eq 1). We calculated in-plane values of ν_xy_ caused by the strain on the unit cell. The expansion ratio was determined by the sum of contributions from the application of the incremental strains as:

$$\varepsilon_{\text{i}}=\;\text{ln}\;\left({\text{L}}_{\text{i}}/{\text{L}}_{0}\right)\;=\;\sum_{\text{i}}^{}\left[\text{ln}\;\left({\text{L}}_{\text{i}}/{\text{L}}_{\text{i}-1}\right)\;+\;\varepsilon_{\text{i}-1}\right]$$ (2)

Where i = 1, 2, 3, 4, …, n denote the current strain state, L_i_ is the specimen length for the current strain state, i and L_0_ is the initial specimen length.

### Cell culture on the construct

Cells were cultured on planar patterns with NPR or PPR unit-cell arrays. To activate the acrylic acid moieties of PEGDA constructs, we incubated them for 2 h in a solution of 0.12 M N-hydoxy succinimide and 0.15 M 1-ethyl-3-(3-dimethylaminopropyl) carbodiimide hydrochloride in 2-(morpholino) ethanesulfonic acid buffer at pH 5. The constructs were rinsed in phosphate buffer saline (PBS) of pH 7.4, then a 2% neutralized atelocollagen solution (Koken Co., Tokyo, Japan) was stratified on the constructs with NPR or PPR unit-cell arrays. These collagen-treated constructs were incubated for 1 h at 37°C, then rinsed with PBS.

For *in vitro* tests, human turbinate mesenchymal stromal cells (hTMSCs, donated by the Catholic University of Korea, obtained from turbinate tissue discarded during turbinate surgery), were cultured in a 5% CO_2_ condition at 37°C in α-MEM containing 1% penicillin/streptomycin and 10% fetal bovine serum (FBS). The cultured hTMSCs were harvested using 0.25% trypsin-ethylenediamine-tetra-acetic acid (Sigma-Aldrich, St. Louis, MO, USA), and seeded on the collagen-treated constructs at 17,000 cells/ml (10 μl cell suspension per sample). The cells seeded onto each construct were permitted to adhere to a surface of the constructs for 2 h, then seeded constructs were cultured in media for 11 d.

### Immunochemical staining

After being cultured for 7 d, cell-seeded constructs were fixed with 10% paraformaldehyde in PBS for 20 min then rinsed with PBS. Fixed samples were immersed in 0.1% Triton-X 100 in PBS for 5 min, then blocked with 0.2% bovine serum albumin (BSA) in PBS for 20 min. The samples were washed with PBS, then incubated in phalloidin-FITC solution (1:100 ratio, Sigma-Aldrich, St. Louis, MO, USA) for 1 h to stain filamentous actin. The samples were briefly washed in PBS to remove unbound antibodies, then mounted on a slide glass with DAPI mounting medium (GBI Labs, WA, USA) to stain the nuclei. Finally, samples were viewed under a FluoView 1000 fluorescence confocal microscope (Olympus Optical, Melville, NY, USA).

### Cell proliferation assay

To observe the cell proliferation on the scaffold, a cell-number counting kit (CCK-8, Dojindo Laboratory, Kumamoto, Japan) was used. First, serum-free α-MEM and CCK-8 solution were mixed 10:1 (v:v). After moving cell cultured scaffolds of day 1, 3 and 7 in a new multi-well plate, then prepared α-MEM with CCK-8 was added to the new well plate. The plates were kept for 4 h in a 5% CO_2_ condition, then the cell proliferation rate was acquired by measuring the absorbance at 450 nm wavelength using a well plate reader (Sunrise Absorbance Reader, TECAN Ltd., Weymouth, UK).

### Statistical analysis

All data are presented as means +/- standard deviation from several separate tests. Data were analyzed by linear regression and one-way analysis of variance (ANOVA) (MINITAB version 14.2, Minitab, State College, PA). When p < 0.05, statistical significance was accepted.

## Results & Discussion

**Fig 2:**
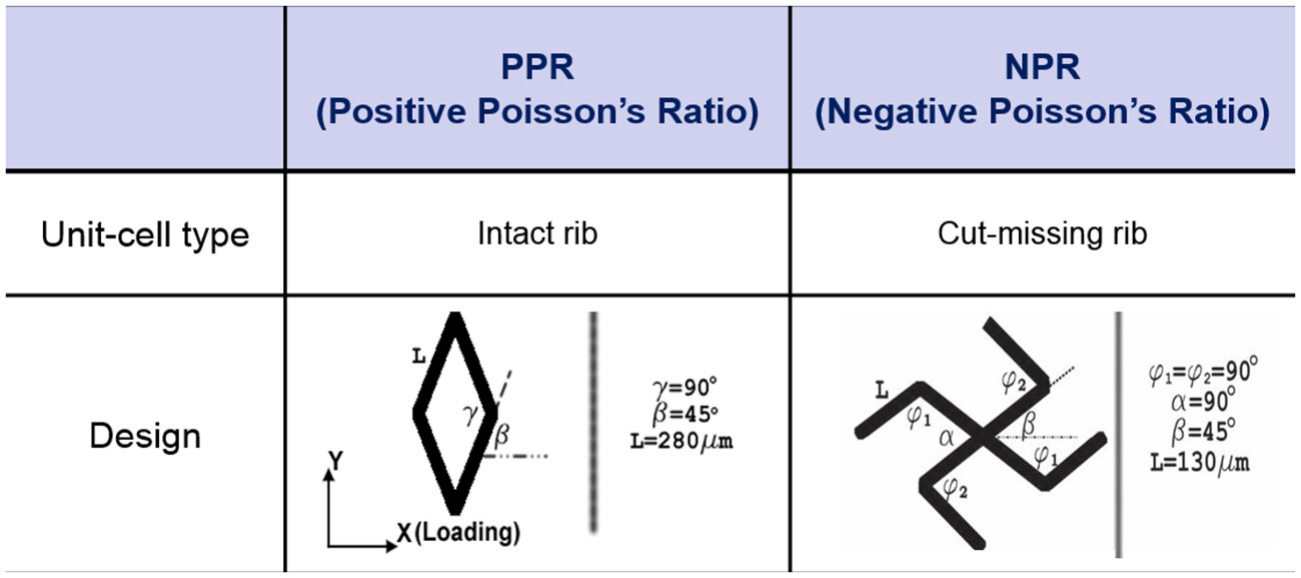
Geometry design of the unit-cells. The walls of the unit-cells (denoted as ribs) are approximately 40 micrometers wide and 100 micrometers deep.

We selected the cut-missing rib unit-cell model to attain the NPR property and an intact-rib unit-cell design for PPR property (Fig 2) [35, 46, 47]. CAD drawings of these constructs were converted into graphic images, which were used as virtual masks for use in fabricating the UV patterns by guiding control of the digital micro-mirror array. Because the cut-missing rib design is formed by removing selected rib shapes from the intact-rib design [47], the cut-missing rib meshwork showed various NPR magnitudes due to the dimensional variation of the structure. For example, changes of the central angles *α* and *β* result in the NPR effect. We designed the original angles as *α* = 90° and *β* = 45°. The intact-rib model was designed in a diamond shape and the properties of this model can be modulated by the values of angles *β* and *γ*, which are coupled naturally to each other in the intact-rib model. This model shows PPR regardless of loading direction. In the strain test for the calculation of ν_xy_, a change of γ was the dominant cause of the deformation. In this study, we set *β* = 45° and *γ* = 90°.

In the unit-cell models (Fig 2), positions and arrangements among rib structures determine the magnitude and sign of ν_xy_; these characteristics are consequences of the combination of flexure, hinging and stretching of designed ribs [48–51]. The unit-cell geometry and the material properties and geometry of the ribs determine the deformation degree of a whole construct. Although ν_xy_ is a strain-dependent value for a cellular material, our results suggest that the shape and orientation of the unit cells have a strong influence on the magnitude and polarity of ν_xy_.

**Fig 3:**
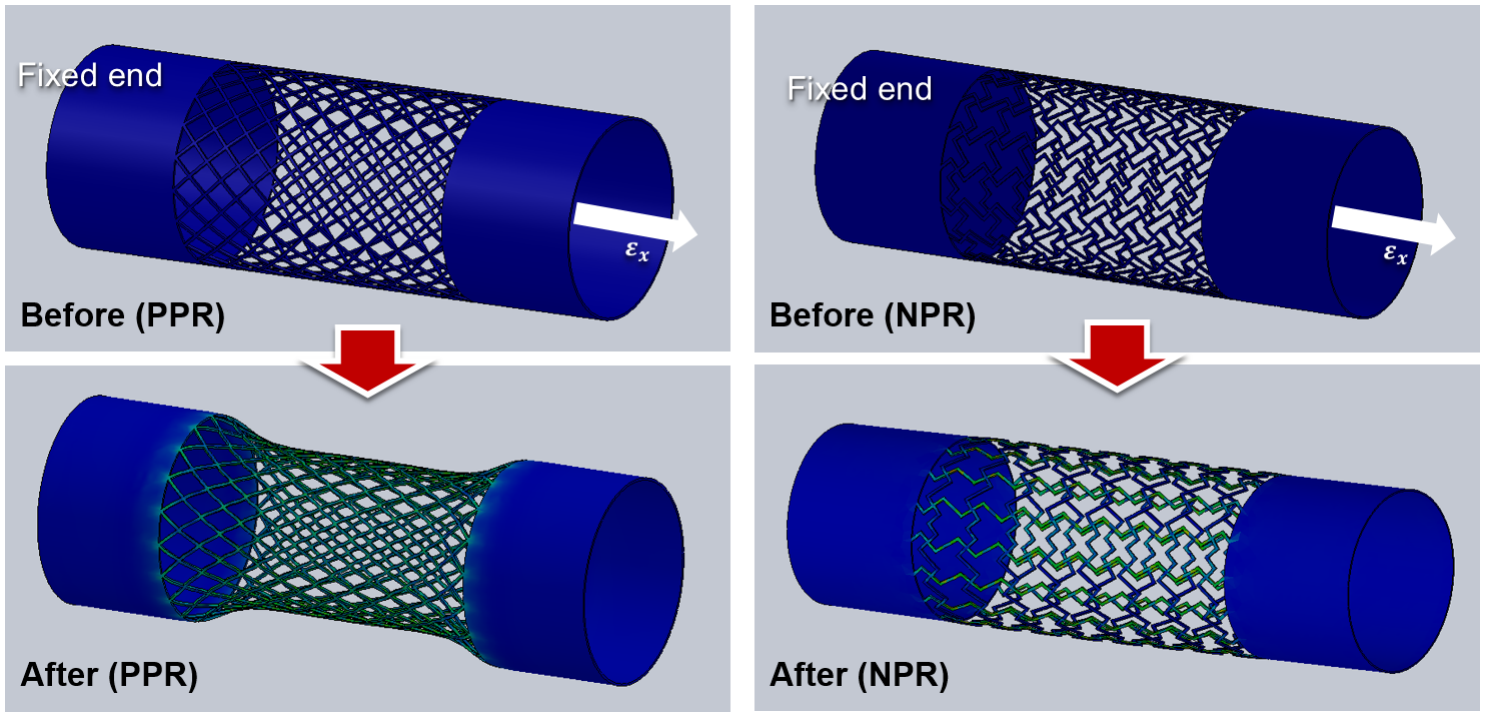
Stress-strain simulations of tubular constructs composed of unit-cells with cut missing-rib and intact-rib architectures.

To determine the deformation mode, we simulated a deformation behavior of the cut-missing and intact rib tubular PEGDA constructs. The simulated deformation resulted from a tensile load of the axial direction (Fig 3). Solid tubular structures were contained both ends of a porous tube to guarantee the mechanical stability of the structure during tensile test. These results indicate that cut-missing rib design may yield NPR behavior in a 3D tubular environment. At the simulation, when the strain of the construct was approximately 0.2, the two kinds of constructs showed the positive and negative Poisson’s ratio as expected. Most deformation was generated by the hinging motion of each unit-cell and the stress was focused on the deformed hinge area. In case of intact rib, we observed that a diameter of the tube was decreased in the middle of the tubular construct and it means that the intact rib construct possesses the positive Poisson’s ratio. In case of cut-missing rib construct, we observed that the construct was slightly extended to the radial direction with the increment of the axial direction. From the calculation, we could conclude that that movement has a negative Poisson’s ratio.

**Fig 4:**
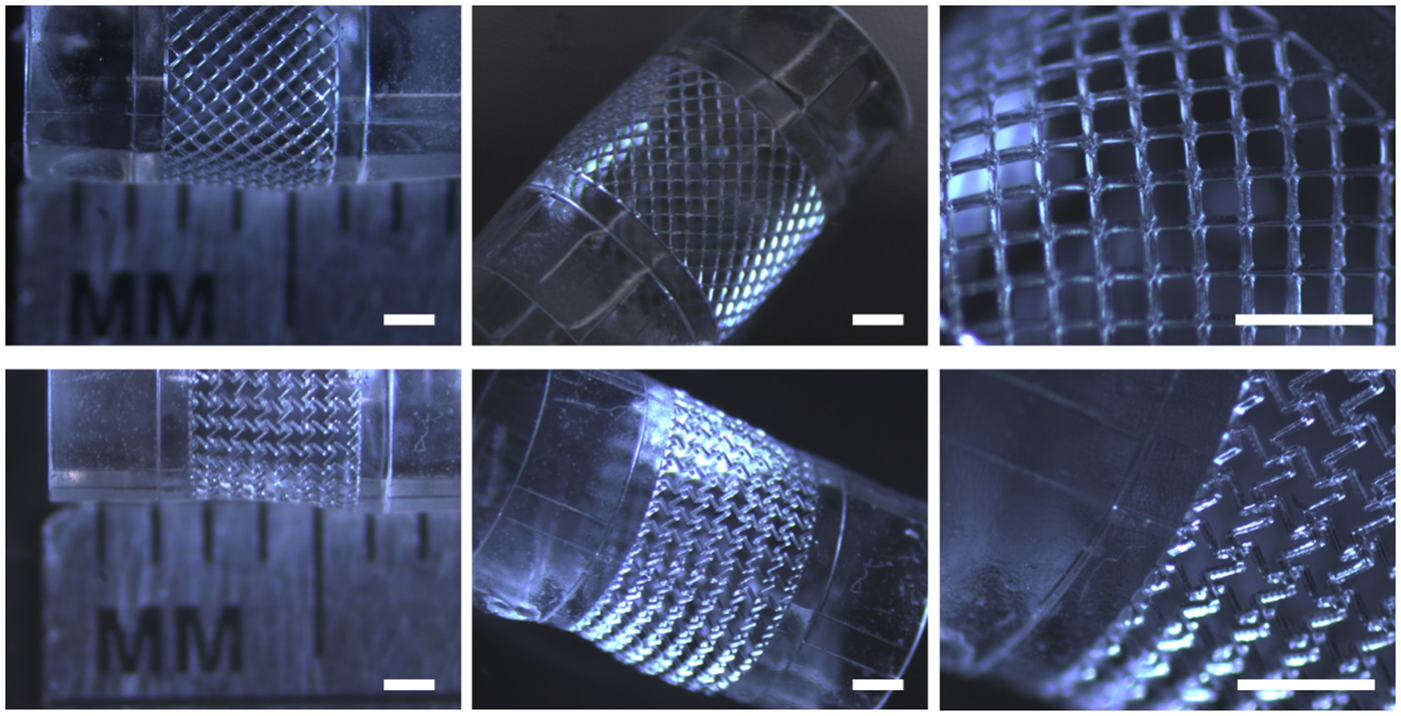
The fabricated tubular constructs. A) PPR construct having intact rib unit-cells. B) NPR construct with cut-missing rib unit-cells.

We successfully fabricated the missing rib and intact rib planar constructs. Using x- and y-axis stage movements, we assembled the pattern array of 3 (column) by 4 (row) from exposures of 12 projection images. The array of unit cells was well-oriented, and enclosed pore geometries by the array of unit-cells were well defined as designed. Rectangular slabs that will be solid supporting tubes at both ends were incorporated at both sides of each construct. Finally, we built the tubular construct by rolling the patterned flat sheet (Fig 4). The tubular constructs had a diameter of ~5 mm.

**Fig 5:**
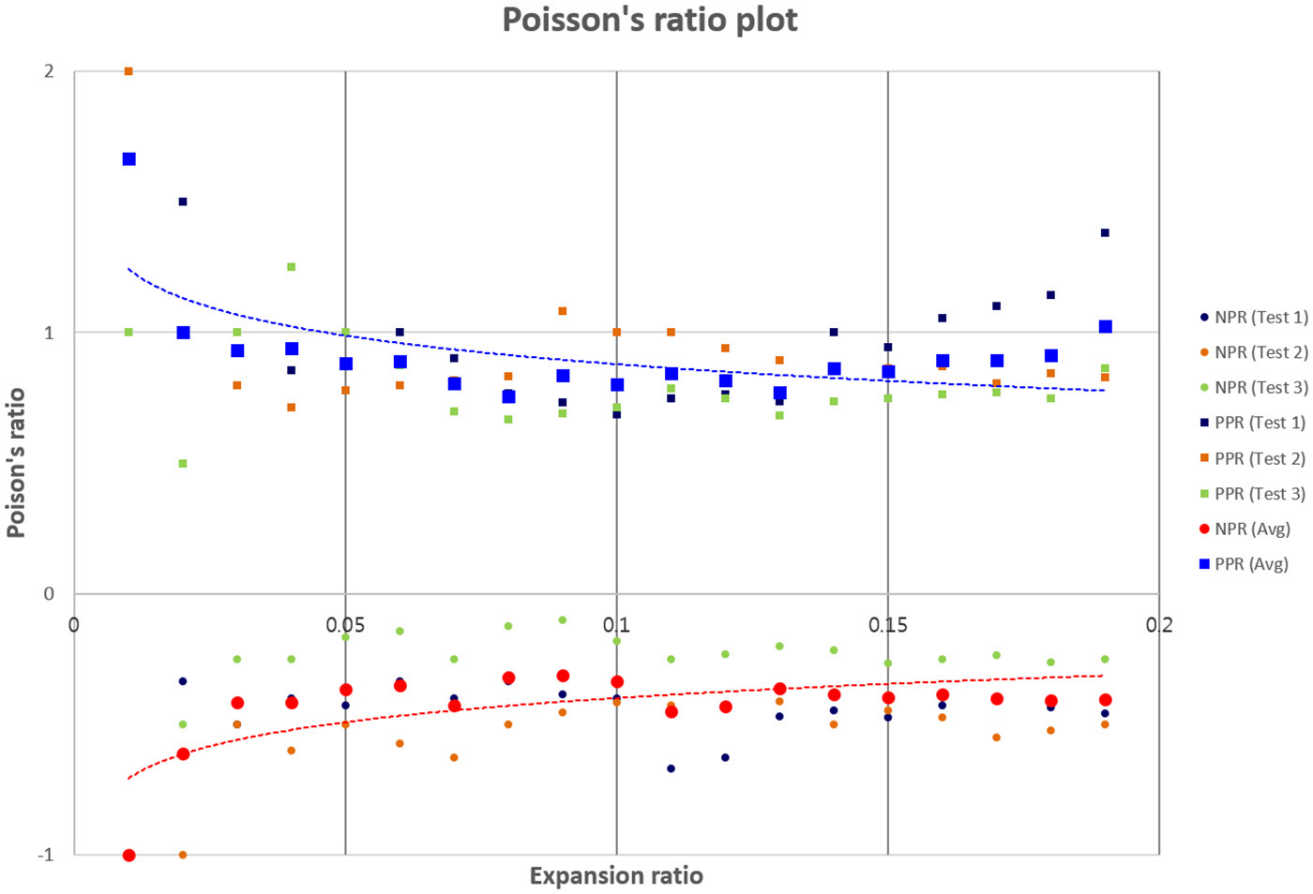
Measured Poisson’s ratio as a function of the expansion ratio for the tubular constructs composed of the cut-missing rib (NPR), and intact rib (PPR) unit-cell geometries. (Three strain-dependent experiments were performed for each type of construct; each strain test was conducted with a different construct. Dark blue: test 1; orange: test 2; green: test 3; red: average of NPR constructs; blue: average of PPR constructs. Circles: NPR constructs; squares, PPR constructs. Dotted red lines: logarithmic fits to the average of NPR constructs; Dotted blue lines: logarithmic fits to the average of PPR constructs.)

**Fig 6:**
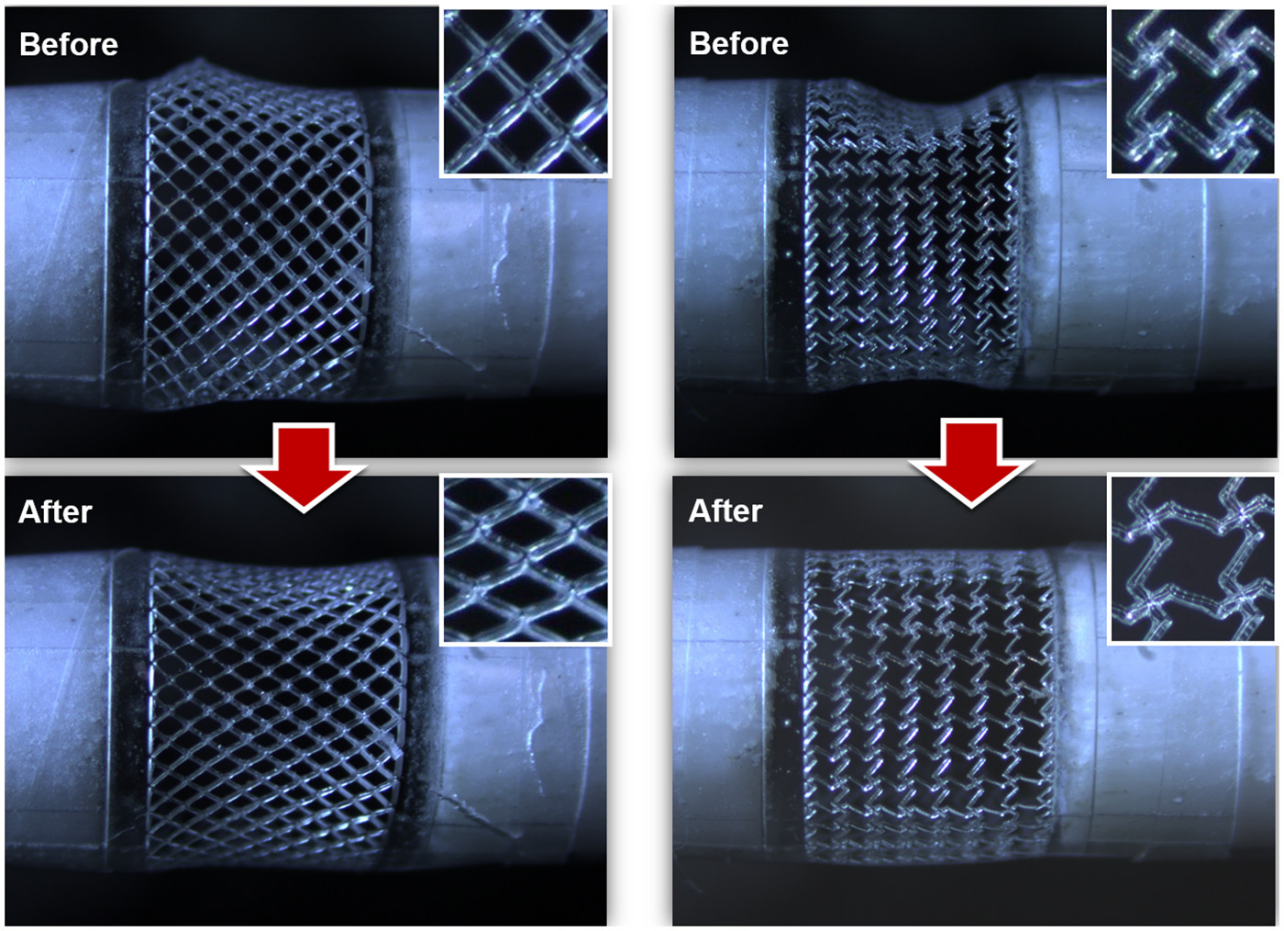
Optical images of the pulling test of PPR and NPR tubular constructs. A) a PPR construct showing a radial contraction and B) an NPR construct demonstrating a radial expansion in the tubular construct.

After fabrication of the tubular construct with unit cells, strain tests were conducted by fixing one end of the tubular construct while applying a tensile load to the other end. ν_xy_ as a function of an instantaneous strain was calculated (Eq 1) by measuring the transverse and axial deformations of unit-cell arrays. ν_xy_ values of NPR and PPR unit-cell were obtained from three constructs each of PPR and NPR constructs for expansion ratios *ε* = δL/L where δL is a change in length, and L is current length; for all constructs, the magnitude of ν_xy_ decreased as ε increased (Fig 5). Optical images (Fig 6) and videos (S1 and S2 Movies) were obtained to observe mechanical responses of tubular constructs caused by the tensile load in the axial direction.

The intact rib constructs with PPR behavior and the cut-missing rib constructs with NPR behavior were tested at the range (0–0.2) of strains. Because PEGDA biomaterial has PPR in itself, our results demonstrated that the unit-cell design induced NPR effect as predicted by the simulation result. In the strain range tested these constructs had -0.6 ≤ ν_xy_ ≤ -0.15, which is similar to theoretical predictions [46]. Normally, Poisson’s ratio is effective for small deformation and linear elasticity. However, because our NPR and PPR constructs showed expected negative and positive Poisson’s ratio behaviors under the axial strain, we think that that our result at the long strain range is reasonable.

The intact rib unit-cell construct showed initial 0.9 ≤ ν_xy_ ≤1.2, which gradually decreased as axial strain increased. The Smith model [46] predicted 0.8 ≤ ν_xy_ ≤ 1 for axial strains of 0 to 0.13. Therefore, our experiment result was similar to that of the theoretical model. Additionally, the deformation of the intact rib (Fig 6) was well matched with the Smith model which holds that the deformation is resulted mainly from the hinging motion of angle γ without stretching of ribs. These results demonstrate that the geometry of unit cells and their arrangement modulate ν_xy_ of the 3D tubular construct.

**Fig 7:**
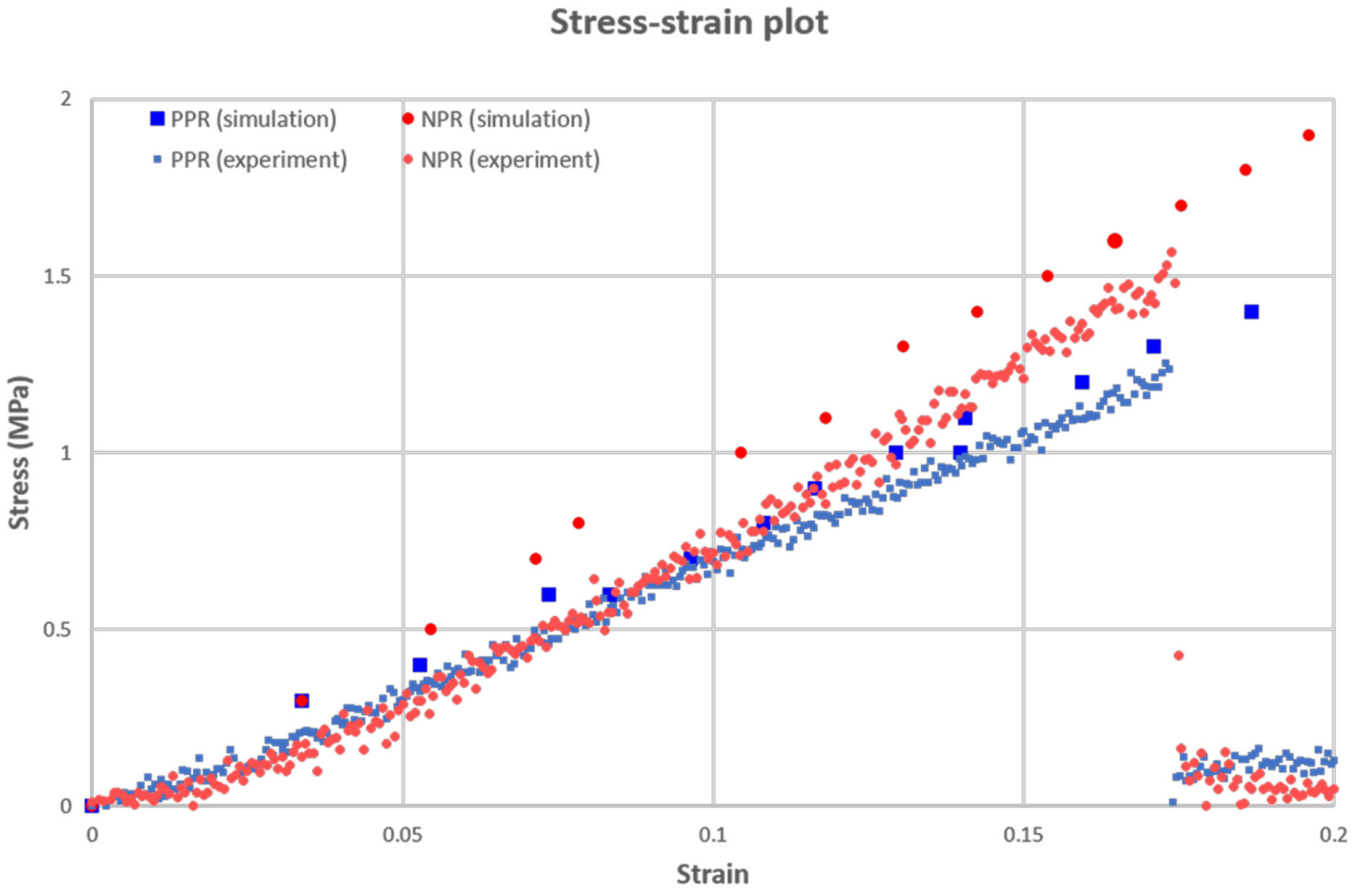
Stress-strain plot of the simulation and experiment at the PPR and NPR constructs. (Large blue-color square: simulation result at PPR construct, Large red-color circle: simulation result at NPR construct, small blue-color square: experiment result at PPR construct, small red-color circle: experiment result at NPR construct.)

To estimate the similarity between the simulation and actual movement, we compared the stress-strain plot of the simulation with that of the tensile experiment. At the simulation, we used the elastic modulus of PEGDA based material reported by previous study [45], we observed the variation of a stress and strain by increasing the tensile load of the tubular construct until the solving process of the simulation is failed. The simulated stress of the NPR construct was higher than that of PPR construct (Fig 7). And calculated elastic moduli of PPR and NPR construct were 7.44 MPa and 9.76 MPa, respectively. Namely, NPR construct showed the higher elastic modulus value than PPR constructs like the stress plot. When we compared moduli of the simulation and experiment, two results showed the similar trends in plot and gaps between them were merely 3 and 7% at PPR and NPR construct, respectively. In addition, experiment values showed the lower than those of simulation, it seems that an actual experiment showed lower flexibility than ideal movement (simulation) because of an immaturity of the hinging motion. And at the experiment, the fractures of the PPR and NPR constructs were observed at the strain of approximately 17% and those values were slightly lower than results of strain tests for the calculation of Poisson’s ratio.

**Fig 8:**
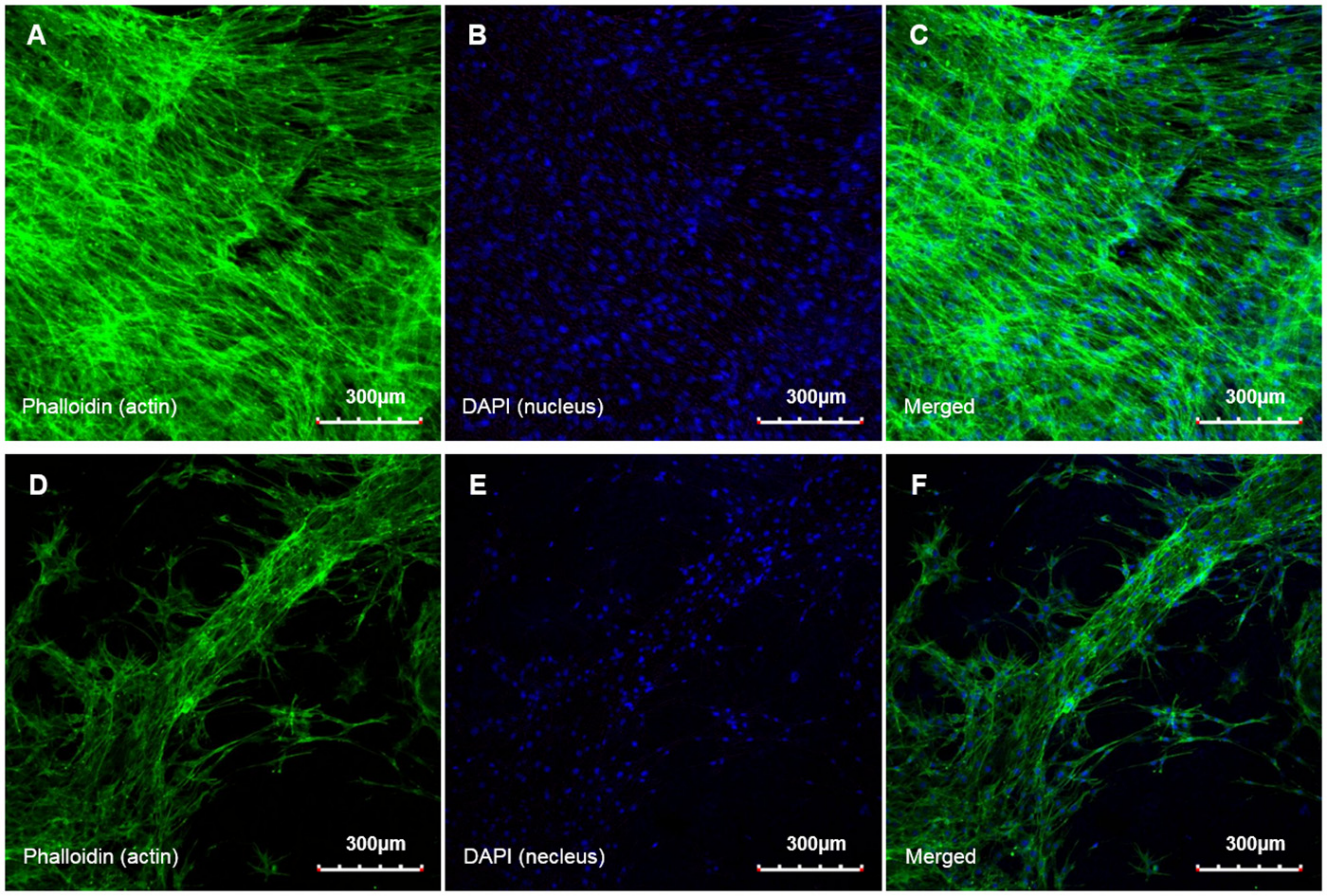
Microscopic images of stained actin filaments (A, D), nuclei (B, E) and merged results (C, F) on the constructs (day 7). A) ~ C) Cut-missing rib (NPR) construct, D) ~ F) Intact rib (PPR) construct.

**Fig 9:**
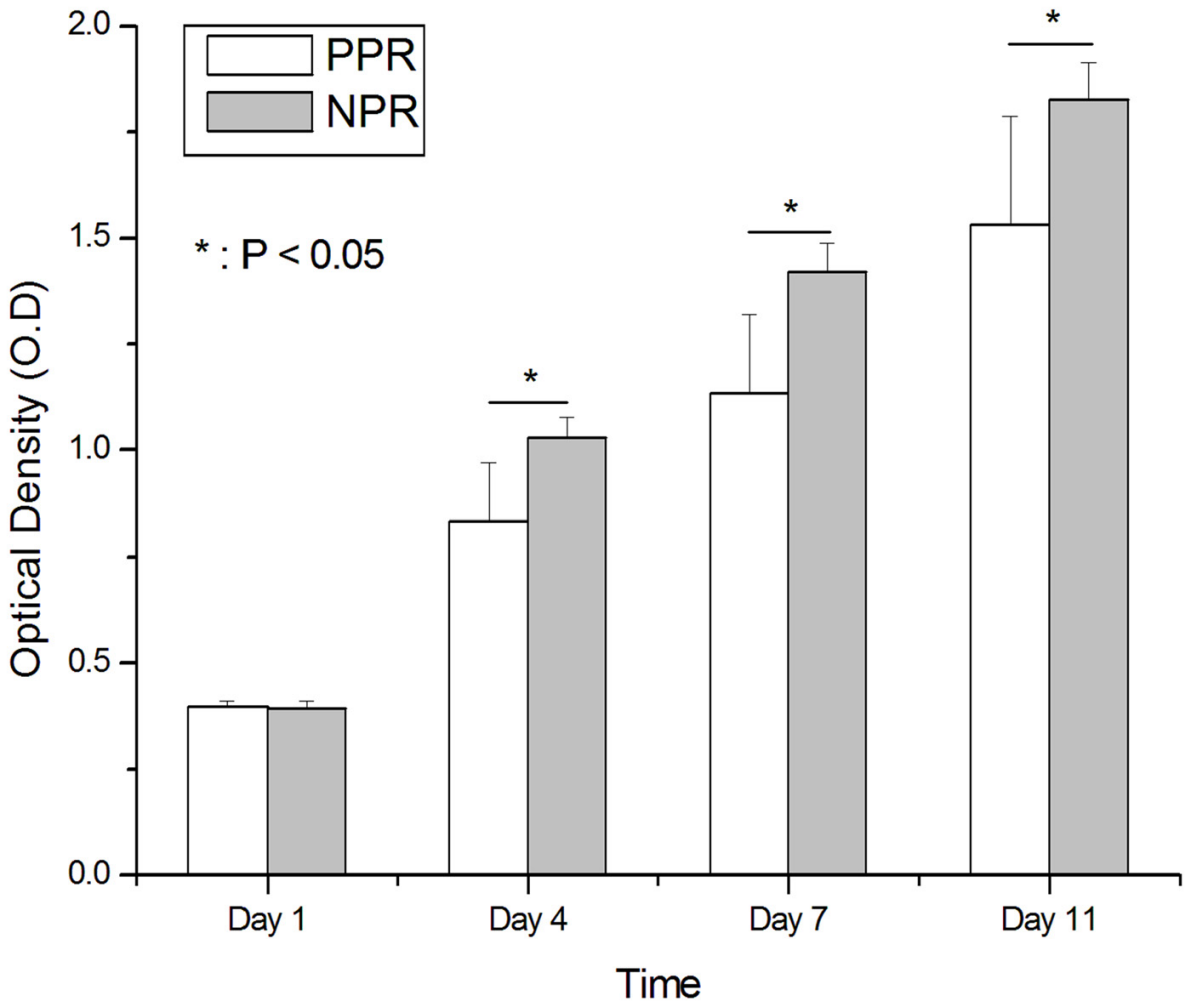
Cell proliferation results on Cut-missing rib (PPR, white) and Intact rib (NRP, grey) constructs. Bars: 1 S.D., n = 6. Asterisks: columns below line are significantly different (p < 0.05)

We also observed attachment and proliferation behaviors of hTMSCs on the developed NPR and PPR tubular constructs. Before the experiment, both types of construct were coated with collagen to promote cellular adhesion. Seeded hTMSCs were attached to the construct struts and actin fibers formed on both the PPR and NPR constructs by 7 d after cell culture (Fig 8). However, in the PPR construct the hTMSCs grew along the construct struts at a partial area, but in the NPR construct they proliferated to the whole area of constructs with a high interconnection as well as along the construct struts. Nuclear staining using DAPI also showed the abundance of the cell on NPR construct. Furthermore, cell proliferation results for 11 d based on the optical density (O.D) measurement by CCK-8 reaction showed the superiority of the NPR construct. Although, an initial cell proliferation rate of day 1 was similar to each other, the NPR construct showed the higher hTMSCs density than the PPR construct at the other days (Fig 9). Although the unit-cell architectures have relatively similar pore size, the spacing among struts was smaller in the NPR unit cell than in the PPR unit cell, so filling by secreted ECM in the pores was easier in the NPR construct than in the PPR construct. Therefore, the cell density was much higher in the NPR construct than in the PPR construct (Fig 8). Those results demonstrate that geometry and arrangement of pores may affect cell attachment behavior.

## Conclusions

We fabricated tubular PEGDA constructs that had either negative (NPR) or positive (PPR) Poisson’s ratios. Planar structures from an analytical model were designed and fabricated into a structure that had NPR. A step-and-print process was conducted to scale up the size of the micron-scale construct to a centimeter-scale sheet, which was then rolled up to form a tube. We also evaluated the cell adhesion by labeling actin filaments and the cell proliferation by measuring optical density. Experiment results confirmed that cell attachment was higher in the constructs that had NPR than in those that had PPR. This work is a significant step toward developing a highly-compliant small-diameter vascular grafts based on NPR materials.

## Supporting Information

S1 Movie A mechanical response of a PPR tubular construct caused by the tensile load in the axial direction. (WMV)

S2 Movie A mechanical response of a NPR tubular construct caused by the tensile load in the axial direction. (WMV)
